# Early Postoperative Rehabilitation Using the Hybrid Assistive Limb (HAL) Lumbar Type in Patients With Hip Fracture: A Pilot Study

**DOI:** 10.7759/cureus.22484

**Published:** 2022-02-22

**Authors:** Tomohiro Fujikawa, Seita Takahashi, Naoki Shinohara, Naohiko Mashima, Masao Koda, Hiroshi Takahashi, Yoshihiro Yasunaga, Yoshiyuki Sankai, Masashi Yamazaki, Kousei Miura

**Affiliations:** 1 Department of Rehabilitation, HITO Medical Center, Shikokuchuo, JPN; 2 Department of Orthopaedics, HITO Medical Center, Shikokuchuo, JPN; 3 Department of Regeneration of Community Medicine, Ehime University Graduate School of Medicine, Toon-shi, JPN; 4 Department of Orthopaedic Surgery, Faculty of Medicine, University of Tsukuba, Tsukuba, JPN; 5 Center for Cybernics Research, University of Tsukuba, Tsukuba, JPN

**Keywords:** exoskeleton, robot rehabilitation, postoperative rehabilitation, hybrid assistive limb, hip fracture

## Abstract

Objective: To extend life expectancy after surgery, patients with hip fractures need to improve their mobility quickly through postoperative rehabilitation. Voluntary hip joint motion supported by the hybrid assistive limb (HAL) lumbar type, an exoskeleton robot suit characterized by its ability to detect the wearer’s intentions through the bioelectrical signals and assist hip extension motions at an optimal timing, may be effective to improve mobility in patients with hip joint dysfunction after surgery. We aimed to introduce rehabilitation using the HAL lumbar type in the early period after hip fracture surgery.

Methods: Patients who underwent internal fixation for hip fracture at a single institution were prospectively enrolled. They received early postoperative rehabilitation (forward and backward bending of the lumbar spine, pelvic tilt forward and backward, standing up, and squatting) using the HAL lumbar type (six times a week for 15 min per session). Five-times-sit-to-stand (FTSS) and timed-up-and-go (TUG) tests were conducted at baseline before HAL rehabilitation (pre-HAL) and after the HAL rehabilitation (post-HAL) intervention.

Results: We enrolled 14 patients (one man, 13 women) in this study. There were no adverse events, and all patients were able to complete the entire rehabilitation program. Post-HAL FTSS showed significant improvement compared with pre-HAL and had a large effect size of 1.81 (95% CI = 0.93 to 2.66) and sufficient power.

Conclusions: Robotic rehabilitation with HAL lumbar type could be introduced without adverse events, even in the early postoperative period following surgery for hip fracture. Further study is needed to develop an appropriate rehabilitation protocol using the HAL lumbar type.

## Introduction

Aging societies have led to increased hip fractures among the elderly worldwide [1]. Hip fracture significantly reduces physical function and quality of life even after surgery [2,3]. Furthermore, the hip fracture has been reported to be a threat to life expectancy [4]. In particular, delayed walking after hip fracture surgery has been associated with poor life expectancy [5]. Therefore, patients who undergo surgery for hip fracture need to improve their mobility quickly through postoperative rehabilitation.

Lee et al. [6] found early mobilization is strongly recommended for rehabilitation after hip fracture surgery. However, rehabilitation procedures after hip fracture surgery have not yet been fully established [7]. Robotic rehabilitation has been used in the postoperative rehabilitation of various diseases. The hybrid assistive limb (HAL; Cyberdyne Inc., Ibaraki, Japan) can estimate the wearer’s motion intention through bioelectrical signals (BES) and provide coordinated joint motion support with appropriate timing and torque. Three types of HAL (lower limb type, single-joint type, lumbar type) are currently being attempted for rehabilitation. There are a number of studies of postoperative rehabilitation using the HAL lower limb type for myelopathy [8,9], cerebral palsy [10], and knee osteoarthritis [11,12]. Rehabilitation using the HAL single-joint type after surgery for knee osteoarthritis has also been reported [13,14]. In this study, we focused on the HAL lumbar type. The HAL lumbar type can detect BES of the lumbar erector spinae muscle and support the wearer’s hip extension movement at optimal timing [15]. It was initially developed as a device to reduce the lumbar load during heavy work and has been reported to reduce the lumbar load during lifting [16], snow-shoveling [17], and simulated patient transfer movements [18]. More recently, it has been considered that support for hip extension movements by the HAL lumbar type may also be effective in supporting the standing movement. To date, robotic rehabilitation using the HAL lumbar type has been attempted for locomotive syndrome [19] and frailty [20]. However, few studies have focused on postoperative rehabilitation in hip fracture patients using the HAL lumbar type. Hip joint motion support by the HAL lumbar type may be effective for hip fracture patients because they have hip joint dysfunction after surgery. This study aimed to introduce rehabilitation using the HAL lumbar type for patients in the early postoperative period after hip fracture surgery.

## Materials and methods

Participants

This study is a pilot, prospective, single-arm, inpatient postoperative rehabilitation study using the HAL lumbar type for patients following hip fracture surgery. Patients who underwent internal fixation for hip fracture at a single institution between April 2019 and June 2020 and satisfied the following inclusion criteria were enrolled: (1) able to walk independently or with a cane before hip fracture; (2) immediate full weight-bearing after surgery was allowed. We excluded the patients who met the following criteria: (1) severe cognitive impairment that makes it difficult to understand the rehabilitation protocol; (2) difficult to walk due to cerebrovascular disease before surgery; (3) implanted pacemakers; (4) underwent bipolar hip arthroplasty. We obtained written informed consent from all participants. This study was conducted following the Declaration of Helsinki with the approval of the Ethical Review Committee of HITO Hospital (Approval No.: 20200107002).

Early postoperative rehabilitation with the HAL lumbar type

**Figure 1:**
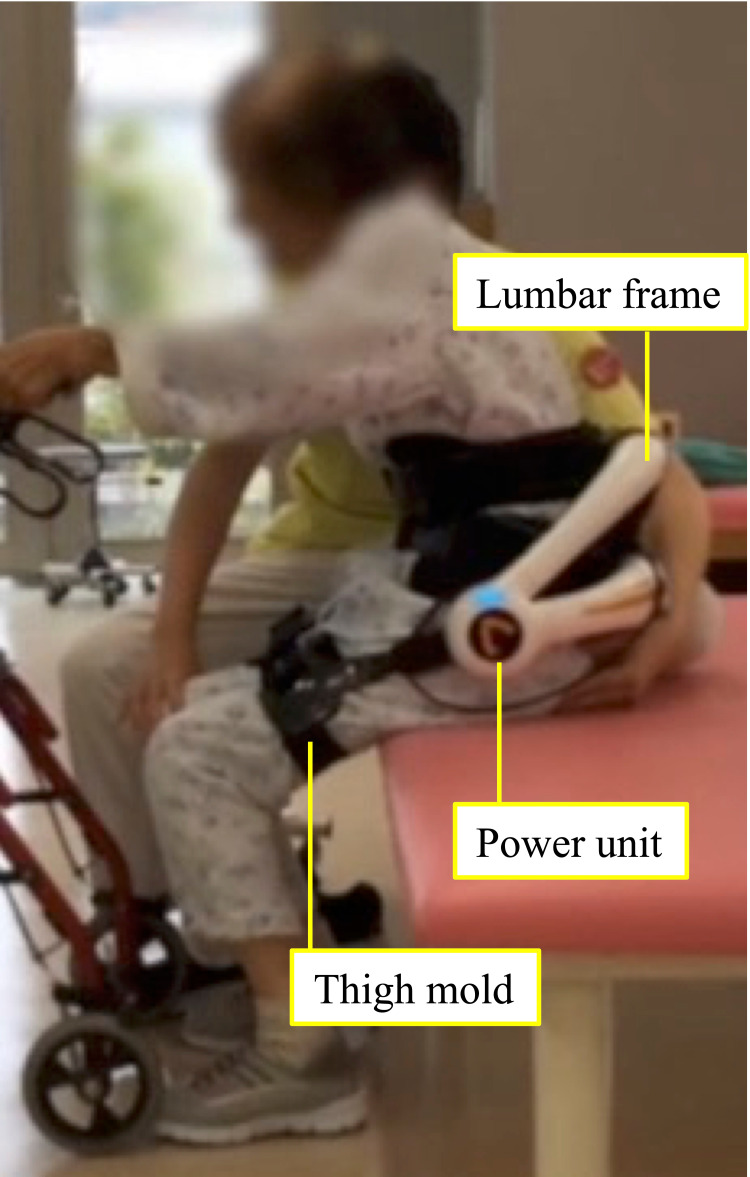
Hybrid assistive limb (HAL) lumbar type The electric motors in the power units can assist hip joint extension motion when a wearer stands up.

The HAL lumbar type consists of a lumbar frame, thigh mold power units, and electrode detecting BES (Figure 1).

It is equipped with angle sensors in the power units located in the bilateral hip joint to detect the wearer’s hip joint angle. The electric motors in the power units can support hip joint extension when the wearer stands up. Based on BES detected from electrodes attached to the lumbar erector spinae muscles, the HAL lumbar type has the characteristic of providing hip joint motion support at the timing intended by the wearer [15].

**Figure 2:**
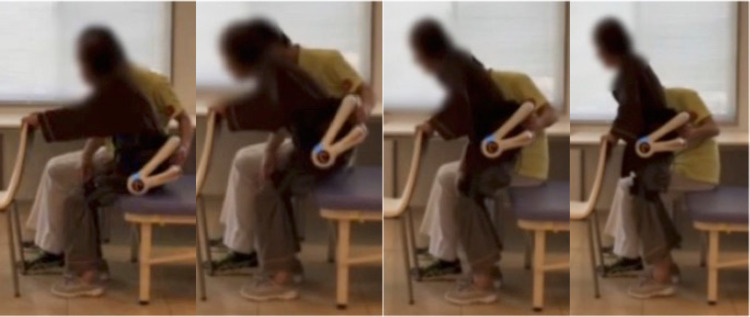
Standing up exercise from a sitting position

**Figure 3:**
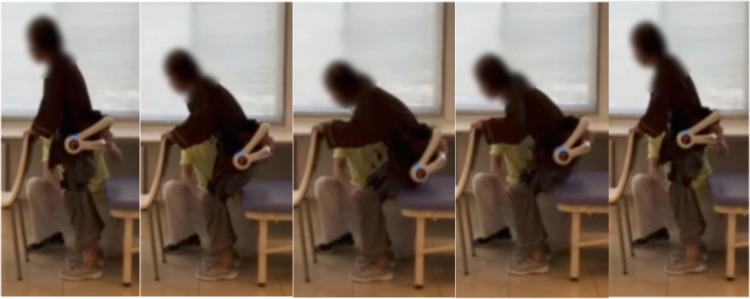
Squatting exercise in a standing position

Participants underwent rehabilitation using the HAL lumbar type in addition to the usual conventional postoperative rehabilitation after hip fracture surgery. Once the patient’s general condition was stabilized after surgery, HAL rehabilitation was started as soon as possible. The following four types of HAL rehabilitation were performed: (1) forward and backward bending of the lumbar spine in a sitting position; (2) pelvic tilt forward and backward in a sitting position; (3) standing up from a sitting position with the support of the upper limbs (Figure 2); (4) squatting in a standing position with the support of the upper limbs (Figure 3).

 

The HAL rehabilitation was conducted six times a week for 15 minutes per session. Rehabilitation using the HAL lumbar type was completed when the patient became able to walk with a walker.

Outcome measures

The five-times-sit-to-stand (FTSS) and the timed-up-and-go (TUG) tests were used to assess locomotion function. These tests were conducted at baseline before HAL rehabilitation (pre-HAL) and after the HAL rehabilitation intervention (post-HAL). The FTSS test was performed at maximum speed while holding onto a 40-cm high platform. The TUG test was performed using a walker both pre-HAL and post-HAL.

A Wilcoxon signed-rank test was used to compare pre-HAL and post-HAL test results. Statistical analysis was performed using the JMP software package version 14.0.0 (SAS Institute, Cary, NC, USA). Effect size and post-hoc statistical power were calculated using Gpower software (version 3.1.9, University of Dusseldorf, Dusseldorf, Germany). P < 0.05 was considered significant.

## Results

Fourteen patients (one man, 13 women) were enrolled in this study. No patient met pre-set exclusion criteria. The demographic data of the participants are summarized in Table 1.

**Table 1:** Patient demographic data FN, femoral neck fracture; FT, femoral trochanteric fracture; HF, hip fracture; HAL, hybrid assistive limb; POD, postoperative day

Characteristics		
Sample size	N (%)	14
Age (years)	Mean ± SD (range)	83.4 ± 8.4 (69-96)
Sex (%)	Male	1 (7.1)
	Female	13 (92.9)
Fracture type (%)	FN	2 (14.3)
	FT	12 (85.7)
Ambulation before HF (%)	Independent	13 (92.9)
	Cane	1 (7.1)
HAL start (POD)	Mean ± SD (range)	5.9 ± 2.5 (2-8)

There were no adverse events associated with rehabilitation using the HAL lumbar type, and all participants were able to complete the entire rehabilitation program. The clinical characteristics of each case are summarized in Table 2.

**Table 2:** Patient clinical characteristics Values are mean ± standard deviation. FN, femoral neck fracture; FT, femoral trochanteric fracture; HF, hip fracture; HAL, hybrid assistive limb; POD, postoperative day; FTSS, five-times-sit-to-stand test; TUG, timed-up-and-go test; *P < 0.01 (compared with Pre-HAL FTSS)

Case	Age (years)	Sex	Fracture type	Ambulation before HF	HAL start (POD)	Walker start (POD)		Pre-HAL FTSS	Post-HAL FTSS		Pre-HAL TUG	Post-HAL TUG
1	72	F	FN	Independent	8	11		16.4	8.22		15.2	8.2
2	85	F	FN	Independent	8	16		35.5	31.4		48.7	25.4
3	85	F	FT	Independent	8	13		41.3	12.33		101.2	18.9
4	70	F	FT	Independent	8	11		32.7	18.2		16.3	11.1
5	91	F	FT	Independent	8	14		42.2	15.1		-	21.4
6	96	F	FT	Independent	8	19		43.2	22.6		-	13.7
7	80	F	FT	Independent	8	12		41.2	21.32		72.0	19.4
8	84	F	FT	Independent	8	10		26.4	20.22		59.7	32.4
9	69	M	FT	Independent	3	7		29.2	6.99		-	14.6
10	83	F	FT	Independent	4	22		39.9	27.84		-	22.9
11	90	F	FT	Independent	4	12		32.4	15.67		-	53.4
12	87	F	FT	Independent	2	7		45.3	25.34		-	24.8
13	94	F	FT	Cane	3	8		38.4	33.72		32.9	29.7
14	81	F	FT	Independent	3	10		24.5	18.24		-	21.6
Mean ± SD								34.9 ± 8.4	19.8 ± 8.0*			
Effect size								1.81 (95 % CI = 0.93-2.66)				
Power								0.99				

Post-HAL FTSS showed significant improvement compared to pre-HAL with a large effect size of 1.81 (95% CI = 0.93 to 2.66) and sufficient power (0.99). In pre-HAL tests, seven out of 14 patients could not walk sufficiently to complete the TUG test, but all patients could achieve it in post-HAL. In all other cases, the TUG time decreased.

## Discussion

In the present study, we aimed to introduce rehabilitation using the HAL lumbar type in the early postoperative period following surgery for a hip fracture. All 14 patients were able to complete the HAL rehabilitation without adverse events. Furthermore, post-HAL FTSS test results showed improvement compared to pre-HAL.

Various rehabilitation procedures after hip fracture surgery have been reported. In a review by Lee et al. [21], they found balance training to positively affect balance, gait, leg strength, and activity in daily life (ADL) after hip fracture surgery. Progressive resistance exercise also has a positive effect on mobility [22]. Early maximum strength training after hip fracture surgery is effective in improving leg strength [23]. By contrast, according to a review by Hulsbæk et al. [24], exercise therapy after hip fracture is not sufficiently effective. Lee et al. reported that physical therapy during the postoperative acute care phase is essential for early discharge, but it is not possible in all cases [6]. That is because fractures, inflammatory agents, and pain associated with surgical invasion occur in the perioperative period following hip fracture surgery and are known to lead to lost opportunities for rehabilitation interventions [25,26]. Therefore, it seems essential to determine a way to assist early rehabilitation intervention after hip fracture surgery.

In the present study, we attempted early postoperative rehabilitation intervention using the HAL lumbar type. To date, several reports have shown that exercise therapy using the HAL lumbar type improved physical functions in the elderly. Kotani et al. [20] reported that five core and squat exercise sessions with the HAL lumbar type for frailty improved motor function, including TUG times. Miura et al. [19] found that balance function improved after 12 sessions of exercise therapy with the HAL lumbar type consisting of sit-to-stand, lumbar flexion-extension, and gait training for the locomotive syndrome. Both studies found that exercise therapy using the HAL lumbar type could be safely performed by elderly patients without exacerbation of pain. A case of exercise therapy for postoperative rehabilitation using HAL lumbar type after lumbar fusion surgery has been reported [27]. To our knowledge, this is the first report of robotic rehabilitation after hip fracture surgery. This study preliminarily introduced the following four types of rehabilitation using the HAL lumbar type: forward and backward bending of the lumbar spine, pelvic tilt forward and backward, standing up and squatting. These exercises appear to increase hip joint function and improve stride length, resulting in increased walking speed. The HAL lumbar type is characterized by its ability to detect the wearer’s intentions through BES and assist hip extension motions at optimal timing. In addition, the motor learning effect induced by interactive biofeedback can provide coordinated joint motion support, which may result in the prevention of overload [28]. Hip fracture occurs mainly in the elderly, so frailty and early postoperative pain may inhibit rehabilitation. For those factors, rehabilitation of hip motion using the HAL lumbar type may be more effective than conventional rehabilitation.

Several limitations of the present study should be considered. The effect of the HAL lumbar type on physical function could not be clarified because there was no comparison with the control group, which received only conventional rehabilitation. It was impossible to prove that the HAL lumbar type reduced the physical load because there was no evaluation of pain and EMG data changes during rehabilitation. Future comparative studies with large sample sizes will be needed to resolve these limitations.

## Conclusions

Robotic rehabilitation with HAL lumbar type could be introduced without adverse events, even in the early postoperative period following internal fixation for hip fracture. Post-HAL FTSS test results showed improvement compared to pre-HAL FTSS test results. These findings suggest that HAL lumbar type may be an option for early postoperative rehabilitation after hip fracture surgery. Further study is needed to demonstrate the effect of HAL lumbar type on physical function and to develop an appropriate rehabilitation protocol.
